# Variation in Photosynthetic Efficiency under Fluctuating Light between *Rose Cultivars* and its Potential for Improving Dynamic Photosynthesis

**DOI:** 10.3390/plants12051186

**Published:** 2023-03-06

**Authors:** Xiao-Qian Wang, Zhi-Lan Zeng, Zi-Ming Shi, Ji-Hua Wang, Wei Huang

**Affiliations:** 1 School of Life Sciences, Northwest University, Xi’an 710069, China; 2 Kunming Institute of Botany, Chinese Academy of Sciences, Kunming 650201, China; 3 University of Chinese Academy of Sciences, Beijing 100049, China; 4 Flower Research Institute of Yunnan Academy of Agricultural Sciences, Kunming 650205, China

**Keywords:** chlorophyll fluorescence, CO_2_ assimilation, mesophyll conductance, photosynthetic limitation, stomatal conductance

## Abstract

Photosynthetic efficiency under both steady-state and fluctuating light can significantly affect plant growth under naturally fluctuating light conditions. However, the difference in photosynthetic performance between different rose genotypes is little known. This study compared the photosynthetic performance under steady-state and fluctuating light in two modern rose cultivars (*Rose hybrida*), “Orange Reeva” and “Gelato”, and an old Chinese rose plant *Rosa chinensis* cultivar, “Slater’s crimson China”. The light and CO_2_ response curves indicated that they showed similar photosynthetic capacity under steady state. The light-saturated steady-state photosynthesis in these three rose genotypes was mainly limited by biochemistry (60%) rather than diffusional conductance. Under fluctuating light conditions (alternated between 100 and 1500 μmol photons m^−2^ m^−1^ every 5 min), stomatal conductance gradually decreased in these three rose genotypes, while mesophyll conductance (*g*_m_) was maintained stable in Orange Reeva and Gelato but decreased by 23% in *R. chinensis*, resulting in a stronger loss of CO_2_ assimilation under high-light phases in *R. chinensis* (25%) than in Orange Reeva and Gelato (13%). As a result, the variation in photosynthetic efficiency under fluctuating light among rose cultivars was tightly related to *g*_m_. These results highlight the importance of *g*_m_ in dynamic photosynthesis and provide new traits for improving photosynthetic efficiency in rose cultivars.

## 1. Introduction

Plants use photosynthesis to convert light energy into stable chemical energy by photosynthetic electron transport and the Calvin-Benson cycle. Plants with high photosynthetic efficiency usually have relatively fast growth rate and high levels of biomass and productivity. The light-saturated photosynthetic capacity under steady state is thought to be the critical determinant of plant growth. For example, the higher steady-state photosynthetic capacity in C_4_ plants facilitates their higher productivity than C_3_ plants under optimal conditions [1,2]. Photosynthesis can be limited by CO_2_ diffusional conductance and biochemical factors [3]. Stomatal conductance (*g*_s_) and mesophyll conductance (*g*_m_) together determine the CO_2_ diffusion from air into chloroplast and thus influence chloroplast CO_2_ concentration [4,5,6,7,8]. Biochemical factors represent the capacity for the Calvin-Benson cycle and photosynthetic electron flow. High values of *g*_s_ and *g*_m_ are the prerequisites of high CO_2_ assimilation rate (*A*_N_) in plants grown under high nitrogen condition and high light [6,8]. Generally, photosynthetic capacity in angiosperms is mainly limited by biochemical factors and *g*_m_ rather than *g*_s_ when measured under favorite conditions [5,9]. However, *g*_m_ imposes the major limitation on *A*_N_ in sclerophyllous oaks [10], *Rhododendron* species [11] and Orchid species [12]. Therefore, the major limiting factor of *A*_N_ might largely differ between species. Modern rose is one of the most important fresh cut flowers all over the world, owing to its high values in ornamental, food and material industry. However, the major limiting factor of light-saturated *A*_N_ under steady state in modern rose cultivars is not well known.

In nature, leaves usually experience fluctuating light due to cloud, wind, and shading from other leaves [13,14]. In addition to steady-state photosynthetic capacity, dynamic photosynthesis under fluctuating light significantly affects plant growth and biomass [15,16,17,18,19]. Upon transitioning from low to high light, net CO_2_ assimilation rate (*A*_N_) gradually increases, but the time required to fulfill light induction largely differs among different plants and cultivars [9,16,18,20,21,22]. For example, C_3_ plants needed less time to accomplish the photosynthetic induction than C_4_ plants [23]. Large variations in the rates of photosynthetic induction were observed in genotypes of African cassava, rice, wheat, and canola [15,18,20,21]. Therefore, improving photosynthetic performance under fluctuating light has a great potential in crop improvement. 

When irradiance sharply increases, photosynthetic induction is tightly related to four steps: (1) the induction rate of photosynthetic electron flow, which can be accomplished in 2 min [19,24]; (2) the activation of ribulose bisphosphate carboxylase/oxygenase (Rubisco), which needs approximately 5–10 min [21,25]; (3) the induction kinetics of *g*_m_, which needs approximately 5–20 min [25,26]; (4) the induction kinetics of *g*_s_, which needs time up to 1 h to reach the maximum value [9,13,18]. Notably, the induction rates of *g*_s_ and *g*_m_ are much slower than those of photosynthetic electron flow and Rubisco. Therefore, in theory, *g*_s_ and *g*_m_ likely exert the major limitations of photosynthesis under fluctuating light [18,26]. Indeed, the induction kinetics of *g*_s_ significantly affected the induction rate of *A*_N_ in *Arabidopsis thaliana* [19], rice [24], and African cassava [18]. A recent study reported that *g*_m_ significantly restricted *A*_N_ during light induction in *Arabidopsis thaliana* and tobacco [26]. Furthermore, the induction of *A*_N_ was more related to *g*_m_ induction rather than *g*_s_ induction in tomato [27]. Therefore, the major limitation of *A*_N_ under fluctuating light differs between species. 

Natural sunlight is the major light source for the cultivation of modern rose cultivars, but their dynamic photosynthesis under fluctuating light is little known. In the breeding of modern rose, some old rose species are usually used as a parent of hybridization, but the photosynthetic characteristics of old rose species are poorly understood. Modern rose cultivars have much higher productivity than old rose plants, but the underlying photosynthetic mechanisms have not yet been clarified. Specifically, it is unclear whether modern rose cultivars have higher photosynthetic capacity under steady state or have superior photosynthetic performance under fluctuating light to old rose species. Based on the results that crop cultivars usually had similar steady-state photosynthesis but varied in dynamic photosynthesis [17], we hypothesize that modern rose cultivars have higher dynamic photosynthetic efficiency than old rose species. 

In the present study, photosynthetic characteristics were measured under steady state and fluctuating light in two modern rose (*Rose hybrida*) cultivars, “Orange Reeva” and “Gelato”, and an old Chinese rose plant *Rosa chinensis*, “Slater’s crimson China”. The aims of this study are: (1) to quantify the limitation of steady-state *A*_N_ in rose cultivars; and (2) to explore whether modern rose cultivars have superior photosynthetic performance under fluctuating light to the old rose germplasm. The results indicated that that photosynthetic capacity under steady state did not differ significantly among these three rose genotypes, and the steady-state photosynthesis was mainly limited by the biochemical capacity in them. However, the two modern *Rose hybrida* cv. “Orange Reeva” and “Gelato” showed stronger photosynthetic performance under fluctuating light than the old germplasm *Rosa chinensis*. Therefore, the improved photosynthetic efficiency under fluctuating light partially contributes to the stronger growth potential of modern rose cultivars. 

## 2. Results

### 2.1. Photosynthetic Characteristics under Steady-State Differ Slightly between Rose Genotypes

**Figure 1:**
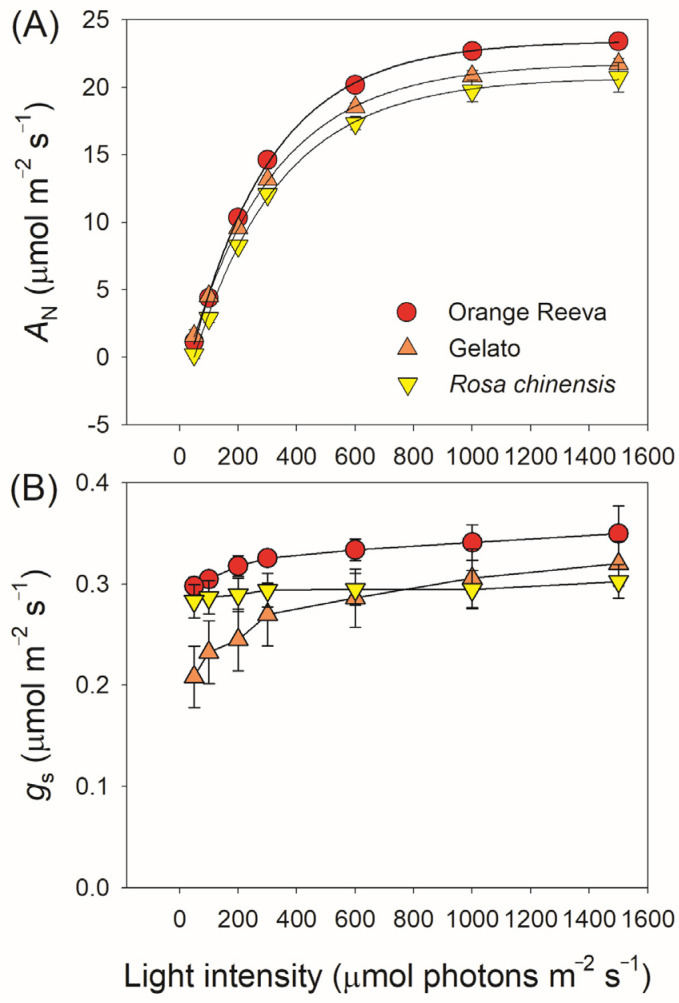
Light intensity dependence of leaf net CO_2_ assimilation rate (*A*_N_) (**A**) and stomatal conductance (*g*_s_) (**B**) in two modern rose cultivars (Orange Reeva and Gelato) and the old Chinese rose plant *Rosa chinensis*. Data are means ± SE (*n* = 5).

The basal leaf functional traits of the three studied rose genotypes were measured and displayed in Table 1. Chlorophyll content (SPAD value) was significantly higher in *Rosa hybrida* cv. Orange Reeva and Gelato than in *Rosa chinensis*. Orange Reeva displayed the highest value of leaf mass per area (LMA), followed by *Rosa chinensis* and Gelato. Leaf N, K, P content in Orange Reeva and Gelato were significantly higher than those in *Rosa chinensis*. At a high light of 1500 μmol m^−2^ s^−1^, values for steady state *A*_N_ were 23.4, 21.7, and 20.7 μmol m^−2^ s^−1^ in Orange Reeva, Gelato, and *Rosa chinensis*, respectively. Concomitantly, no significant difference in *g*_s_ was observed among these three rose genotypes, but Orange Reeva and Gelato had significantly higher *g*_m_ than *Rosa chinensis*. Dark respiration rate (*R*_d_) did not significantly differ among these rose genotypes, while the maximum rate of RuBP carboxylation (*V*_cmax_) was significantly higher in Orange Reeva than Gelato and *Rosa chinensis*. Generally, the light response curves indicated that these three rose genotypes showed similar *A*_N_ and *g*_s_ at a given light intensity (Figure 1). Therefore, the steady-state photosynthesis differed only slightly among different rose genotypes. 

**Figure 2:**
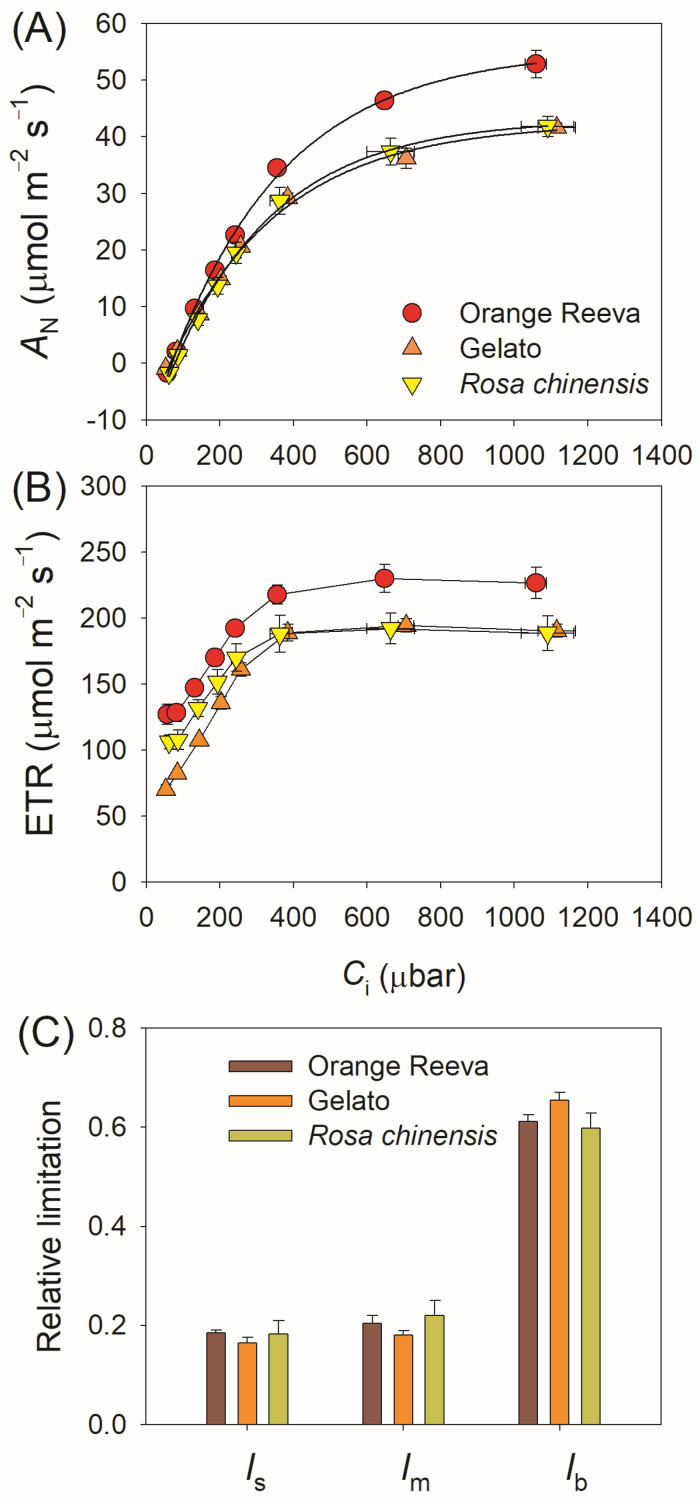
Response of leaf net CO_2_ assimilation rate (*A*_N_; **A**) and electron transport rate (ETR; **B**) to intracellular CO_2_ concentration in two modern rose cultivars (Orange Reeva and Gelato) and the old Chinese rose plant *Rosa chinensis*. (**C**) Quantitative analysis of relative limitation imposed on *A*_N_ in these three rose genotypes. *l*_s_, stomatal conductance limitation, *l*_m_, mesophyll conductance limitation, and *l*_b_, biochemistry limitation. All *A*/*C*_i_ curves were measured under a saturating light of 1500 μmol photons m^−2^ s^−1^. Data are means ± SE (*n* = 5).

Based on the CO_2_ response curves, *A*_N_ differed very slightly between these three rose genotypes at *C*_i_ below 300 μmol mol^−1^ (Figure 2A). However, when *C*_i_ was higher than 300 μmol mol^−1^, *Rosa hybrida* cv. Orange Reeva had significantly higher *A*_N_ than *Rosa hybrida* cv. Gelato and *Rosa chinensis* (Figure 2A). Concomitantly, electron transport rate through PSII (*J*_PSII_) was higher in Orange Reeva than the other two rose genotypes (Figure 2B). At an atmospheric CO_2_ concentration of 400 μmol mol^−1^, *A*_N_ just reached 40–50% of the maximum value, but *J*_PSII_ reached approximately 80% of the maximum value (Figure 2A,B). Therefore, the major limitation imposed on *A*_N_ at 1500 μmol m^−2^ s^−1^ and 400 μmol mol^−1^ CO_2_ was Rubisco carboxylation rather than RuBP regeneration (i.e., electron transport rate). The quantitative analysis indicated that the relative limitation imposed on *A*_N_ by biochemical capacity was approximately 0.6 in the three rose genotypes, the relative limitation of *g*_s_ or *g*_m_ was approximately 0.2 in them (Figure 2C). Therefore, in the three studied rose genotypes, biochemistry was the major limitation of *A*_N_ under atmospheric CO_2_ concentration and high light, followed by diffusional conductance. 

### 2.2. Modern Rose Cultivars Use Fluctuating Light More Efficiently Than the Old Rose Species

**Figure 3:**
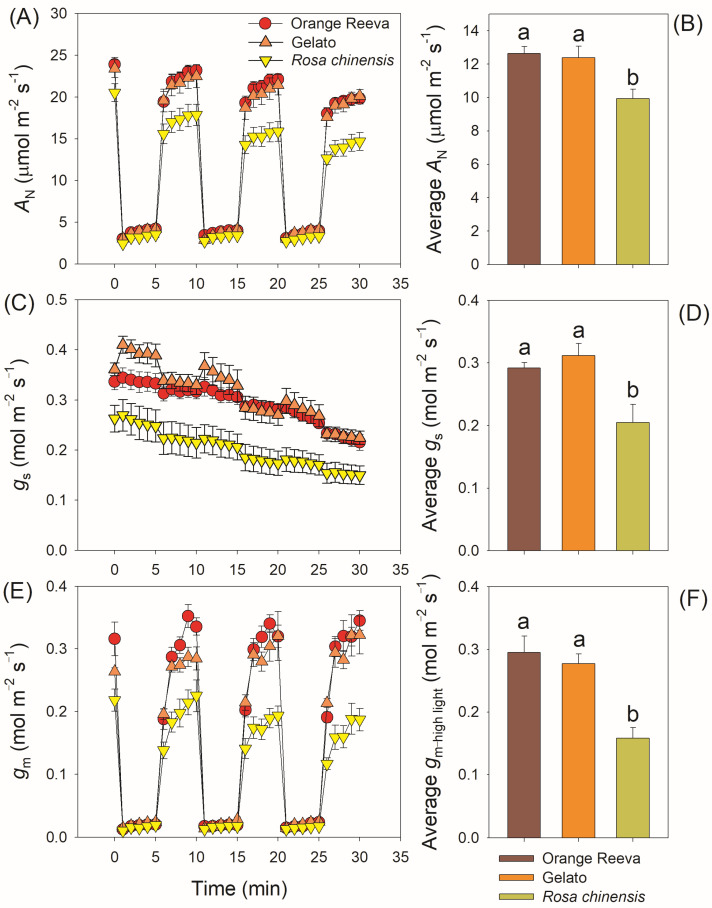
Dynamic changes and average values of leaf net CO_2_ assimilation rate (*A*_N_) (**A**,**B**), stomatal conductance (*g*_s_) (**C**,**D**), and mesophyll conductance (*g*_m_) (**E**,**F**) under fluctuating light in two modern rose cultivars (Orange Reeva and Gelato) and the old Chinese rose plant *Rosa chinensis*. Adapted leaves were exposed to four repeated cycles of 100 and 1500 μmol photons m^−2^ s^−1^ (every 5 min). Data are means ± SE (*n* = 5).

**Figure 4:**
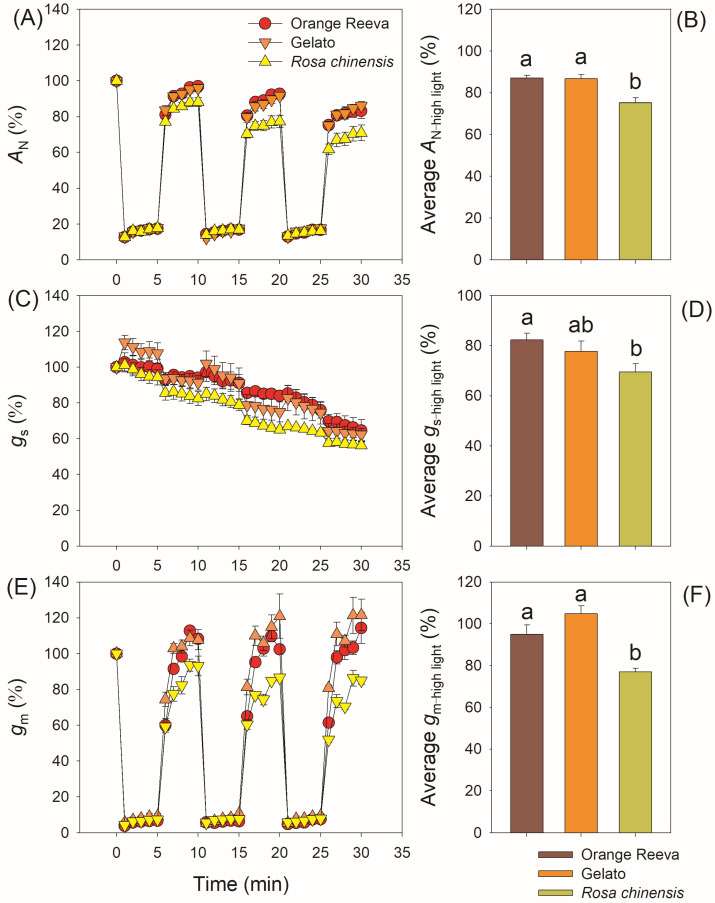
Relative changes and average values of in leaf net CO_2_ assimilation rate (*A*_N_) (**A**,**B**), stomatal conductance (*g*_s_) (**C**,**D**), and mesophyll conductance (*g*_m_) (**E**,**F**) under fluctuating light in two modern rose cultivars (Orange Reeva and Gelato) and the old Chinese rose plant *Rosa chinensis*. Adapted leaves were exposed to four repeated cycles of 100 and 1500 μmol photons m^−2^ s^−1^ (every 5 min). Relative values were calculated as the percentage of the initial steady-state value. Data are means ± SE (*n* = 5).

**Figure 5:**
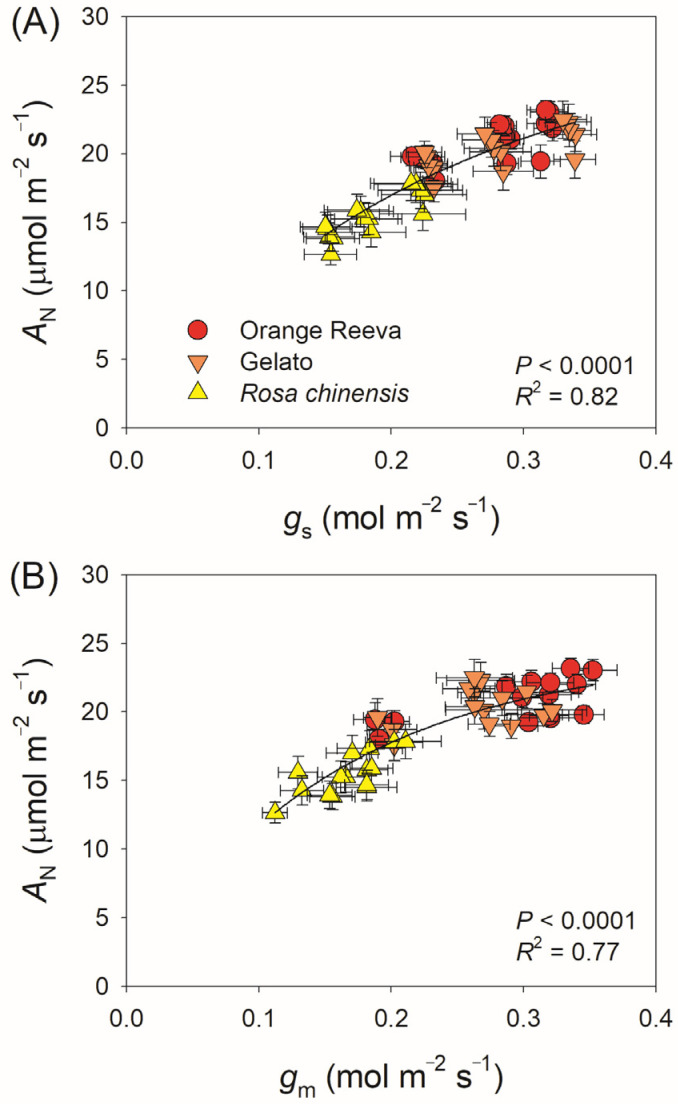
Relationships between stomatal conductance (*g*_s_) and leaf net CO_2_ assimilation rate (*A*_N_) (**A**) and between mesophyll conductance (*g*_m_) and *A*_N_ (**B**) during high light phase in fluctuating light. Data are means ± SE (*n* = 5).

During the three low/high light cycles, Orange Reeva and Gelato had significantly higher *A*_N_ in high-light phases than *Rosa chinensis*, while the value of *A*_N_ in low-light phases did not differ between them (Figure 3A). Such difference in *A*_N_ in high-light phases led to the higher carbon gain under fluctuating light in Orange Reeva and Gelato (Figure 3B). During the 30 min fluctuating light treatment, *g*_s_ gradually decreased with prolonged illumination under fluctuating light in all these three rose genotypes (Figure 3C), and the average *g*_s_ under fluctuating light was significantly higher in Orange Reeva and Gelato than *Rosa chinensis* (Figure 3D). Upon transitioning to high light, *g*_m_ gradually increased in the subsequent 5 min (Figure 3E). No significant difference in *g*_m_ was observed at low light, while Orange Reeva and Gelato had significantly higher *g*_m_ at high-light phases than *Rosa chinensis* (Figure 3F). When normalized to the initial values, *Rosa chinensis* displayed significant lower *A*_N_, *g*_s_, and *g*_m_ under high-light phases than Orange Reeva and Gelato (Figure 4). Therefore, the two modern *Rose hybrida* cultivars use fluctuating light more efficiently than the old rose genotype *Rosa chinensis*. Furthermore, tight relationships between *A*_N_ and diffusional conductance (*g*_s_ and *g*_m_) were observed (Figure 5), suggesting that the relatively lower photosynthetic efficiency under fluctuating light in *Rosa chinensis* was partially attributed to its lower *g*_s_ and *g*_m_. 

**Figure 6:**
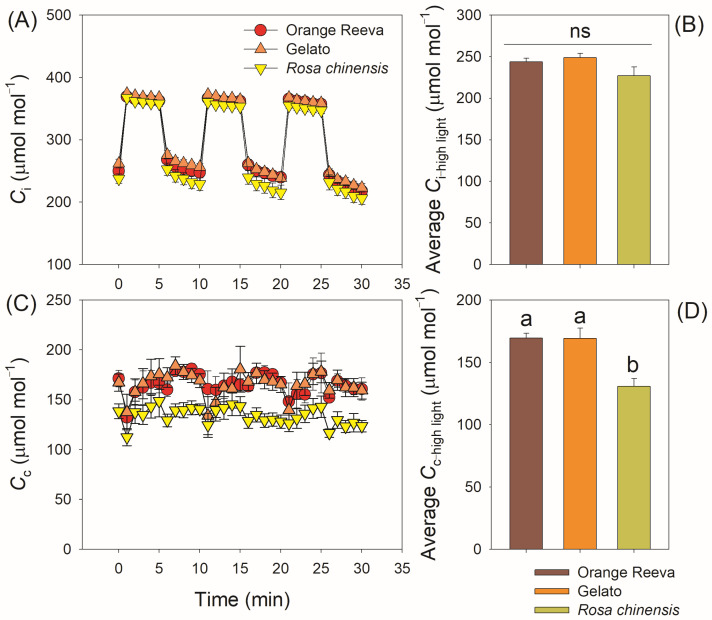
Dynamic changes and average values of intercellular CO_2_ concentration (*C*_i_) (**A**,**B**) and chloroplast CO_2_ concentration (*C*_c_) (**C**,**D**) under fluctuating light in two modern rose cultivars (Orange Reeva and Gelato) and the old Chinese rose plant *Rosa chinensis*. Adapted leaves were exposed to four repeated cycles of 100 and 1500 μmol photons m^−2^ s^−1^ (every 5 min). Data are means ± SE (*n* = 5).

**Figure 7:**
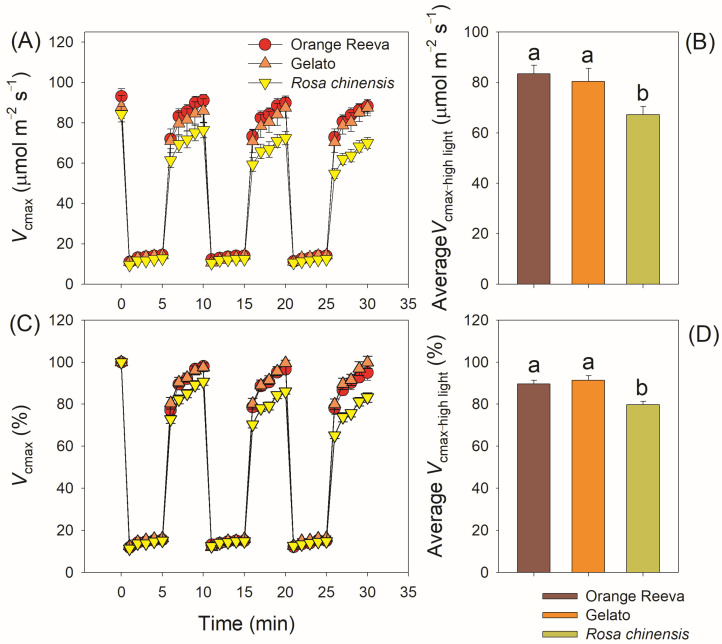
Dynamic changes (**A**), relative changes (**C**), and average values (**B**,**D**) of the maximum velocity of Rubisco carboxylation (*V*_cmax_) in two modern rose cultivars (Orange Reeva and Gelato) and the old Chinese rose plant *Rosa chinensis*. Adapted leaves were exposed to four repeated cycles of 100 and 1500 μmol photons m^−2^ s^−1^ (every 5 min). Relative values were calculated as the percentage of the initial steady-state value. Data are means ± SE (*n* = 5).

During fluctuating light treatment, *C*_i_ did not significantly differ among these three rose genotypes (Figure 6A). However, the *C*_c_ values under high-light phases were significantly higher in Orange Reeva and Gelato than *Rosa chinensis* (Figure 6B). Under steady-state photosynthesis at high light, these three rose genotypes had similar value of *V*_cmax_ (Figure 7A). After exposure to the three cycles of low/high light, *V*_cmax_ could increase to the initial value after 5 min illumination at high light in Orange Reeva and Gelato but remarkedly decreased in *Rosa chinensis* (Figure 7A), making the average *V*_cmax_ under high light in *Rosa chinensis* was lower than the other two genotypes (Figure 7B). By normalizing to the initial steady-state value, *V*_cmax_ decreased to a much lower extent in *Rosa chinensis* when compared with Orange Reeva and Gelato (Figure 7A,B). These results indicated that the difference in *A*_N_ under fluctuating light between different rose genotypes was correlated to *C*_c_ and *V*_cmax_ rather than *C*_i_. 

## 3. Discussion

In general, the major limiting factor of photosynthesis largely varied among different species or different genotypes of a given species. Alternating the relative limitation imposed on photosynthesis at the leaf level can improve plant biomass and crop productivity [19,28,29,30]. The relative limitation of steady-state photosynthesis under saturating light has been investigated in many crops and groups [5,9]. However, leaves rarely conduct steady-state photosynthesis when exposed to natural sunlight [31,32,33]. While exploring the major limitation under steady state is valuable for understanding photosynthetic regulation, dynamic photosynthetic measurements provide insight into how crop leaves respond to fluctuating light and has great potential in crop improvement [14,18,21]. As showed in Figure 1, the steady-state photosynthesis changed slightly among the three rose cultivars. However, the dynamic photosynthetic efficiency under fluctuating light was significantly higher in two modern rose cultivars Orange Reeva and Gelato when compared with the old rose plant *Rosa chinensis* (Figure 3), providing important new trait for the modern rose cultivars. Therefore, improving dynamic photosynthesis under fluctuating light is a potential target for increasing rose yield. 

### 3.1. Steady-State Photosynthesis across Rose Germplasm Is Mainly Limited by Biochemical Capacity 

Despite some uncertainties regarding the methods for *g*_m_ estimation, the quantitative analysis indicated that the limitation to steady-state photosynthesis imposed by *g*_m_ or *g*_s_ in all three rose genotypes was approximately 20% (Figure 2C). Therefore, increasing *g*_s_ and *g*_m_ might have minor roles in improving light-saturated photosynthesis under steady state in the breeding of rose cultivars. Concomitantly, the relative limitation imposed on *A*_N_ by biochemistry was approximately 60% (Figure 2C), indicating that biochemical capacity was the major limitation imposed on photosynthesis at steady state in these three rose genotypes. This characteristics of photosynthetic limitation in rose plants were similar to herbaceous plants, such as rice [9] and tomato [27], but different from sclerophyllous angiosperms, such as evergreen Mediterranean oaks [10] and *Rhododendron* species [11]. 

At the atmospheric CO_2_ concentration of 400 μmol mol^−1^, photosynthetic electron transport reached 80–90% of the maximum value while *A*_N_ just reached 40–50% of the maximum value (Figure 2). Therefore, biochemical limitation was mainly attributed to Rubisco activity in vivo rather than regeneration of RuBP. On average, *V*_cmax_ in the three studied rose genotypes was 108 μmol m^−2^ s^−1^, which was low when compared to elite cultivars of wheat and rice [34,35]. *V*_cmax_ estimated by *A*/*C*_i_ curve is tightly determined by Rubisco content and efficiency, suggesting that rose genotypes grown under similar conditions of good nutrient might have relatively lower Rubisco content and/or efficiency than other high-yield C_3_ crops. This difference in *V*_cmax_ suggests that strategies proposed to improve Rubisco quantity and efficiency would have particular value in improving steady-state photosynthetic rate [36,37,38]. Therefore, increasing Rubisco content and activity through genetic manipulation might significantly increase yield potential in rose genotypes, which should be taken into consideration in molecular breeding of rose cultivars.

### 3.2. Modern Rose cultivars have Stronger Dynamic Photosynthetic Efficiency Than the Old Rose Rosa chinensis

The loss of photosynthetic carbon gain under fluctuating light can significantly affect plant growth and biomass [15,19,31,39]. During fluctuating light treatment with low/high light cycles, the decline of *A*_N_ under high light was observed in the three studied rose cultivars (Figure 3A and Figure 4A), which was similar to the phenomenon of *Arabidopsis*, rice, and tomato. Such loss of photosynthetic carbon gain in rose genotypes was particularly caused by the gradual decrease in *g*_s_ under fluctuating light (Figure 5). Previous studies indicated that improved induction speed of *g*_s_ or increased *g*_s_ under fluctuating light significantly increased photosynthetic efficiency and biomass in *Arabidopsis thaliana* and rice when grown under fluctuating light [14,15,19]. Similarly, the decline in *g*_s_ is a common photosynthetic characteristic in rose genotypes when exposed to fluctuating light, indicating that increasing *g*_s_ or altering the response of *g*_s_ to change of light intensity is an attractive target for improving photosynthetic efficiency under fluctuating light in this crop. 

In modern rose cultivars Orange Reeva and Gelato, the gradual decrease in *g*_s_, not the change of *g*_m_, accounted for the declines in *A*_N_ under fluctuating light (Figure 4). By comparison, the decline in *A*_N_ under fluctuating light in old rose cultivar *Rosa chinensis* was caused by the simultaneous decreases in *g*_s_ and *g*_m_ (Figure 4). Therefore, the underlying mechanisms for the decline in *A*_N_ are different between different cultivars. Previous studies mainly focused on the effect of stomatal behavior on dynamic photosynthesis among different crop germplasms [16,17,18,40]. However, little attention is given to the behavior of *g*_m_ under fluctuating light and its effect on photosynthetic carbon loss. Some recent studies reported that *g*_m_ can exert a significant limitation of photosynthesis under fluctuating light [26,27]. Once light intensity abruptly increased, the induction speed of *g*_m_ was rapider in Orange Reeva and Gelato than in *Rosa chinensis*. This different response of *g*_m_ to fluctuating light led to significant higher *C*_c_ and *V*_cmax_ values in Orange Reeva and Gelato (Figure 6 and Figure 7), which facilitated the higher efficiency of dynamic photosynthesis in them. Therefore, the response kinetics of *g*_m_ significantly affect the photosynthetic efficiency under fluctuating light across rose germplasm. An improved kinetics of *g*_m_ can favor photosynthesis under fluctuating light, which is an attractive strategy for the breeding of high-yield cultivars of other horticultural plants and crops. 

## 4. Materials and Methods

### 4.1. Plant Materials and Growth Conditions

Two industrial *Rosa hybrida* cv. “Orange Reeva” and “Gelato” and an old Chinese rose plant *Rosa chinensis* cv. “Slater’s crimson China” were used. These plants were cultivated in a greenhouse located in Kunming, Yunnan, China, with 50% full sunlight, day and night air temperatures of 35 and 20 °C, respectively, and relative air humidity of 45–60%. The maximum light intensity to which the leaves were exposed was approximately 1000 μmol photons m^–2^ s^–1^. Plants were watered and fertilized (0.1% nutrient solution) every day. The uppermost mature leaves on the flower stems were chosen for measurements. 

### 4.2. Gas Exchange and Chlorophyll Fluorescence Measurements

Gas exchange and chlorophyll fluorescence were measured simultaneously using an open gas exchange system (LI-6400XT; Li-Cor Biosciences, Lincoln, NE, USA) equipped with a leaf chamber fluorometer (Li-Cor Part No. 6400–40, enclosed leaf area: 2 cm^2^) at leaf temperature of 25 °C, a relative humidity of approximately 60%, and air flow rate of 300 mmol min^–1^. Irradiance was provided by a mixture of red (90%) and blue (10%) LEDs in the fluorometer. After fully induction at 1500 μmol photons m^–2^ s^–1^, light response curves were measured under different light intensity (1500, 1000, 600, 300, 200, 100, 50 μmol photons m^–2^ s^–1^), and CO_2_ response curves were measured at each CO_2_ concentration (50, 100, 200, 300, 400, 600, 800, 1000 and 1500 μmol mol^−1^). In light and CO_2_ response curves, photosynthetic parameters were logged after upon reaching steady-state conditions (at least 3 min). The maximum rates of RuBP carboxylation (*V*_cmax_) and regeneration (*J*_max_) were calculated using the *A*/*C*_i_ curves [41]. Dynamic photosynthesis was measured under fluctuating light alternating between low light (100 μmol photons m^–2^ s^–1^; 5 min) and high light (1500 μmol photons m^–2^ s^–1^; 5 min). During three cycles of low/high light, photosynthetic parameters were logged every minute to calculate the kinetics of photosynthesis under fluctuating light.

Chlorophyll fluorescence parameters were determined using the multi-phase flash (MPF) protocol following recommended procedures [42]. The measuring light intensity and the maximum flash intensity were 1 and 8000 μmol m^−2^ s^−1^, respectively. The flash intensity decreased by 60% during the second phase of the MPF and the durations of the three flash phases were 0.3 s, 0.7 s, and 0.4 s, respectively. The effective photochemistry quantum yield of photosystem II (ΦPSII) and total electron transport rate through PSII (*J*_PSII_) were calculated using following equations [43,44]:

$$\Phi_{\mathrm{PSII}}=\frac{\left(F_{m}’-F_{s}\right)}{F_{m}’}\;J_{\mathrm{PSII}}=\Phi_{\mathrm{PSII}}\times \mathrm{PPFD}\times s$$

where *F*_s_ and *F*_m_′ are steady and maximum fluorescence under actinic light, respectively; PPFD is the light intensity, *s* is a unitless lumped calibration factor used to scale Φ_PSII_ to *J*_PSII_ [45], and a typical value of 0.45 was used in this study.

### 4.3. Calculations of g_m_, C_c_ and V_cmax_

Based on the concurrent measurements of *A*_N_ and *J*_PSII_, *g*_m_ was calculated using the following equation [46]:

$$g_{m}=\frac{A_{N}}{C_{i}-\Gamma^{*}\left(J_{\mathrm{PSII}}+8\left(A_{N}+R_{d}\right)\right)/\left(J_{\mathrm{PSII}}-4\left(A_{N}+R_{d}\right)\right)}$$

where *A*_N_ represents the net CO_2_ assimilation rate; *C*_i_, intercellular CO_2_ concentration; Γ^*^, CO_2_ compensation point in the absence of daytime respiration [47,48], and a typical value of 40 μmol mol^–1^ was used in this study. *R*_d_, respiration rate in the dark and was considered to be half of the mitochondrial respiration rate as measured after dark adaptation for 10 min [5]. The chloroplast CO_2_ concentration (*C*_c_) was calculated using the values of *A*_N_*, C*_i_ and *g*_m_ [41,49]:

$$C_{c}=C_{i}-\frac{A_{N}}{g_{m}}$$

The maximum rate of Rubisco carboxylation (*V*_cmax_) was calculated as described by [48,50].

$$V_{\mathrm{cmax}}=\frac{\left(A_{N}+R_{d}\right)\left(C_{i}+K_{m}\right)}{\left(C_{i}-\Gamma^{*}\right)}$$

where *K*_m_ is the effective Rubisco Michaelis–Menten constant for CO_2_ under 21% O_2_ [50,51].

### 4.4. Quantitative Limitation Analysis of A_N_

Factors limiting steady-state photosynthesis in the studied species were also assessed. *l*_s_ represents the relative photosynthetic limitation of *g*_s_; *l*_m_ represents the relative photosynthetic limitation of *g*_m_; *l*_b_ represents the relative photosynthetic limitation of biochemistry. The values of *l*_s_, *l*_m_ and *l*_b_ were calculated using the following equations [3]:

$$l_{s}=\frac{g_{\mathrm{tot}}/g_{s}\times \partial A_{N}/\partial C_{c}}{g_{\mathrm{tot}}+\partial A_{N}/\partial C_{c}}\;l_{m}=\frac{g_{\mathrm{tot}}/g_{m}\times \partial A_{N}/\partial C_{c}}{g_{\mathrm{tot}}+\partial A_{N}/\partial C_{c}}\;l_{b}=\frac{g_{\mathrm{tot}}}{g_{\mathrm{tot}}+\partial A_{N}/\partial C_{c}}$$

where *g*_tot_ was the total CO_2_ diffusional conductance and was calculated as 1/*g*_tot_ = 1/*g*_s_ +1/*g*_m_ [3], and ∂*A*_N_/∂*C*_c_ was calculated according to the methods of [9,48].

$$\partial A_{N}/\partial C_{c}=V_{c,\max}\frac{\Gamma^{*}+K_{c}\left(1+O/K_{o}\right)}{{\left(C_{c}+K_{c}\left(1+O/K_{o}\right)\right)}^{2}}$$

where *K*_c_ and *K*_o_ are the Rubisco Michaelis–Menten constants for CO_2_ and O_2_, respectively, and O is the oxygen concentration in the chloroplasts [48]. 

### 4.5. SPAD Index and Leaf Nutrient Content Measurements

The relative content of chlorophyll per unit leaf area (SPAD index) was measured using a SPAD-502 Plus (Minolta, Tokyo, Japan). After detached from plants, leaf area was measured using a LI-3000A (Li-Cor, Lincoln, NE, USA). Subsequently, these detached leaf samples were dried at 80 °C for 48 h, and dry weight was measured to calculate leaf mass per area (LMA). Finally, leaf N, P, K content was measured using a Vario MICRO Cube Elemental Analyzer (Elementar Analysensysteme GmbH, Langenselbold, Germany). 

### 4.6. Statistical Analysis

Five independent leaves from five different plants were used for each measurement. One-way ANOVA was used to examine the significant differences between different rose cultivars (*α* = 0.05). 

Average values ± SE (*n* = 5) are shown for leaf chlorophyll content (SPAD), leaf mass per area (LMA), leaf N content, leaf K content, leaf P content, net assimilation rate (*A*_N_), stomatal conductance (*g*_s_), mesophyll conductance (*g*_m_), dark respiration rate (*R*_d_), the maximum velocity of Rubisco carboxylation (*V*_cmax_), and regeneration (*J*_max_). Steady-state values of *A*_N_, *g*_s_ and *g*_m_ were measured at 1500 μmol photons m^−2^ s^−1^ as indicated in light response curves. *V*_cmax_ and *J*_max_ were calculated from CO_2_ response curves. Different letters (a, b and c) indicate significant differences between different cultivars.

## 5. Conclusions

The results presented in this study highlight the main traits of the photosynthetic characteristics of rose cultivars under steady state and under fluctuating light. First, Rubisco activity is the major limiting factor of photosynthesis under steady state in rose cultivars, suggesting that increasing Rubisco activity might improve photosynthesis in this crop. Second, the decline in *g*_s_ is an important reason for the loss of photosynthesis under fluctuating light in these three rose cultivars, pointing out that increasing *g*_s_ is a potential target for improvement of photosynthetic efficiency under fluctuating light. Third, the rapid response kinetics of *g*_m_ is a prerequisite of the high photosynthetic efficiency under fluctuating light in modern rose cultivars. Taking together, increasing Rubisco activity has large potential in improvement of photosynthetic efficiency in rose genotypes, which could be strengthened by improving the response kinetics of *g*_s_ and *g*_m_ under fluctuating light. 

## Figures and Tables

**Table 1:** Photosynthetic characteristics of three studied rose genotypes. Different letters at the end of values indicate significant difference among these three cultivars.

Parameters	Orange Reeva	Gelato	*Rosa chinensis*
SPAD	51.4 ± 0.48 a	53.1 ± 0.81 a	46.9 ± 0.39 b
LMA (g m^−2^)	58.04 ± 2.3 a	48.0 ± 0.83 b	53.02 ± 0.93 c
Leaf N content (mg/g)	43.2 ± 0.72 a	40.5 ± 1.4 a	26.4 ± 1.7 c
Leaf K content (mg/g)	22.2 ± 0.7 a	22.3 ± 0.92 a	12.4 ± 0.30 b
Leaf P content (mg/g)	5.76 ± 0.06 a	5.08 ± 0.04 b	3.35 ± 0.18 c
*A*_N_ (μmol m^−2^ s^−1^)	23.9 ± 0.4 a	21.7 ± 0.4 b	20.7 ± 1.1 b
*g*_s_ (mol m^−2^ s^−1^)	0.35 ± 0.03 a	0.32 ± 0.02 a	0.30 ± 0.02 a
*g*_m_ (mol m^−2^ s^−1^)	0.31 ± 0.03 a	0.27 ± 0.03 a	0.19 ± 0.02 b
*R*_d_ (μmol m^−2^ s^−1^)	1.18 ± 0.04 a	1.00 ± 0.04 a	1.22 ± 0.06 a
*V*_cmax_ (μmol m^−2^ s^−1^)	123 ± 5.6a	97.5 ± 2.7b	98.6 ± 4.4b
*J*_max_ (μmol m^−2^ s^−1^)	130 ± 8.0a	99.8 ± 4.7b	101 ± 4.5b

## Data Availability

All relevant data are included in the paper.

## References

[1] Yamori W., Hikosaka K., Way D.A. (2014). Temperature response of photosynthesis in C3, C4, and CAM plants: Temperature acclimation and temperature adaptation. Photosynth. Res..

[2] Sage R.F., Kubien D.S. (2007). The temperature response of C3 and C4 photosynthesis. Plant Cell Environ..

[3] Grassi G., Magnani F. (2005). Stomatal, mesophyll conductance and biochemical limitations to photosynthesis as affected by drought and leaf ontogeny in ash and oak trees. Plant Cell Environ..

[4] Flexas J., Niinemets Ü., Gallé A., Barbour M.M., Centritto M., Diaz-Espejo A., Douthe C., Galmés J., Ribas-Carbo M., Rodriguez P.L. (2013). Diffusional conductances to CO_2_ as a target for increasing photosynthesis and photosynthetic water-use efficiency. Photosynth. Res..

[5] Carriquí M., Cabrera H.M., Conesa M., Coopman R.E., Douthe C., Gago J., Gallé A., Galmés J., Ribas-Carbo M., Tomás M. (2015). Diffusional limitations explain the lower photosynthetic capacity of ferns as compared with angiosperms in a common garden study. Plant Cell Environ..

[6] Yamori W., Nagai T., Makino A. (2011). The rate-limiting step for CO_2_ assimilation at different temperatures is influenced by the leaf nitrogen content in several C3 crop species. Plant Cell Environ..

[7] Flexas J., Díaz-Espejo A., Conesa M.A., Coopman R.E., Douthe C., Gago J., Gallé A., Galmés J., Medrano H., Ribas-Carbo M. (2016). Mesophyll conductance to CO_2_ and Rubisco as targets for improving intrinsic water use efficiency in C3 plants. Plant Cell Environ..

[8] Campany C.E., Tjoelker M.G., von Caemmerer S., Duursma R.A. (2016). Coupled response of stomatal and mesophyll conductance to light enhances photosynthesis of shade leaves under sunflecks. Plant Cell Environ..

[9] Xiong D., Douthe C., Flexas J. (2018). Differential coordination of stomatal conductance, mesophyll conductance, and leaf hydraulic conductance in response to changing light across species. Plant. Cell Environ..

[10] Peguero-Pina J.J., Sisó S., Flexas J., Galmés J., García-Nogales A., Niinemets Ü., Sancho-Knapik D., Saz M.Á., Gil-Pelegrín E. (2017). Cell-level anatomical characteristics explain high mesophyll conductance and photosynthetic capacity in sclerophyllous Mediterranean oaks. New Phytol..

[11] Huang W., Yang Y.-J., Wang J.-H., Hu H. (2019). Photorespiration is the major alternative electron sink under high light in alpine evergreen sclerophyllous Rhododendron species. Plant Sci..

[12] Yang Z.-H., Huang W., Yang Q.-Y., Chang W., Zhang S.-B. (2018). Anatomical and diffusional determinants inside leaves explain the difference in photosynthetic capacity between Cypripedium and Paphiopedilum, Orchidaceae. Photosynth. Res..

[13] Pearcy R.W. (1990). Sunflecks and photosynthesis in plant canopies. Annu. Rev. Plant Physiol. Plant Mol. Biol..

[14] Slattery R.A., Walker B.J., Weber A.P.M., Ort D.R. (2018). The impacts of fluctuating light on crop performance. Plant Physiol..

[15] Adachi S., Tanaka Y., Miyagi A., Kashima M., Tezuka A., Toya Y., Kobayashi S., Ohkubo S., Shimizu H., Kawai-Yamada M. (2019). High-yielding rice Takanari has superior photosynthetic response to a commercial rice Koshihikari under fluctuating light. J. Exp. Bot..

[16] Tanaka Y., Adachi S., Yamori W. (2019). Natural genetic variation of the photosynthetic induction response to fluctuating light environment. Curr. Opin. Plant Biol..

[17] Acevedo-Siaca L.G., Coe R., Wang Y., Kromdijk J., Quick W.P., Long S.P. (2020). Variation in photosynthetic induction between rice accessions and its potential for improving productivity. New Phytol..

[18] De Souza A.P., Wang Y., Orr D.J., Carmo-Silva E., Long S.P. (2020). Photosynthesis across African cassava germplasm is limited by Rubisco and mesophyll conductance at steady state, but by stomatal conductance in fluctuating light. New Phytol..

[19] Kimura H., Hashimoto-Sugimoto M., Iba K., Terashima I., Yamori W. (2020). Improved stomatal opening enhances photosynthetic rate and biomass production in fluctuating light. J. Exp. Bot..

[20] Liu J., Zhang J., Estavillo G.M., Luo T., Hu L. (2021). Leaf N content regulates the speed of photosynthetic induction under fluctuating light among canola genotypes (*Brassica napus* L.). Physiol. Plant..

[21] Salter W.T., Merchant A.M., Richards R.A., Trethowan R., Buckley T.N. (2019). Rate of photosynthetic induction in fluctuating light varies widely among genotypes of wheat. J. Exp. Bot..

[22] Soleh M.A., Tanaka Y., Nomoto Y., Iwahashi Y., Nakashima K., Fukuda Y., Long S.P., Shiraiwa T. (2016). Factors underlying genotypic differences in the induction of photosynthesis in soybean [*Glycine max* (L.) Merr.]. Plant. Cell Environ..

[23] Li Y.-T., Luo J., Liu P., Zhang Z.-S. (2021). C4 species utilize fluctuating light less efficiently than C3 species. Plant Physiol..

[24] Yamori W., Kusumi K., Iba K., Terashima I. (2020). Increased stomatal conductance induces rapid changes to photosynthetic rate in response to naturally fluctuating light conditions in rice. Plant. Cell Environ..

[25] Sakoda K., Yamori W., Groszmann M., Evans J.R. (2021). Stomatal, mesophyll conductance, and biochemical limitations to photosynthesis during induction Research Article. Plant Physiol..

[26] Liu T., Barbour M.M., Yu D., Rao S., Song X. (2022). Mesophyll conductance exerts a significant limitation on photosynthesis during light induction. New Phytol..

[27] Sun H., Zhang Y.-Q., Zhang S., Huang W. (2022). Photosynthetic Induction Under Fluctuating Light Is Affected by Leaf Nitrogen Content in Tomato. Front. Plant Sci..

[28] Kromdijk J., Głowacka K., Leonelli L., Gabilly S.T., Iwai M., Niyogi K.K., Long S.P. (2016). Improving photosynthesis and crop productivity by accelerating recovery from photoprotection. Science.

[29] South P.F., Cavanagh A.P., Liu H.W., Ort D.R. (2019). Synthetic glycolate metabolism pathways stimulate crop growth and productivity in the field. Science.

[30] Simkin A.J., López-Calcagno P.E., Raines C.A. (2019). Feeding the world: Improving photosynthetic efficiency for sustainable crop production. J. Exp. Bot..

[31] Zhu X.G., Ort D.R., Whitmarsh J., Long S.P. (2004). The slow reversibility of photosystem II thermal energy dissipation on transfer from high to low light may cause large losses in carbon gain by crop canopies: A theoretical analysis. J. Exp. Bot..

[32] Taylor S.H., Long S.P. (2017). Slow induction of photosynthesis on shade to sun transitions in wheat may cost at least 21% of productivity. Philos. Trans. R. Soc. B Biol. Sci..

[33] Papanatsiou M., Petersen J., Henderson L., Wang Y., Christie J.M., Blatt M.R. (2019). Optogenetic manipulation of stomatal kinetics improves carbon assimilation, water use, and growth. Science.

[34] Masumoto C., Ishii T., Hatanaka T., Uchida N. (2005). Mechanism of High Photosynthetic Capacity in BC 2 F 4 LinesDerived from a Cross between Oryza sativa and Wild Relatives *O. rufipogon*. Plant Prod. Sci..

[35] Driever S.M., Lawson T., Andralojc P.J., Raines C.A., Parry M.A.J. (2014). Natural variation in photosynthetic capacity, growth, and yield in 64 field-grown wheat genotypes. J. Exp. Bot..

[36] Parry M.A.J., Madgwick P.J., Carvalho J.F.C., Andralojc P.J. (2007). Paper Presented at International Workshop on Increasing Wheat Yield Potential, Cimmyt, Obregon, Mexico, 20–24 March 2006 Prospects for increasing photosynthesis by overcoming the limitations of Rubisco. J. Agric. Sci..

[37] Whitney S.M., Houtz R.L., Alonso H. (2011). Advancing Our Understanding and Capacity to Engineer Nature’s CO_2_-Sequestering Enzyme, Rubisco. Plant Physiol..

[38] Carmo-Silva E., Scales J.C., Madgwick P.J., Parry M.A.J. (2015). Optimizing Rubisco and its regulation for greater resource use efficiency. Plant. Cell Environ..

[39] Ohkubo S., Tanaka Y., Yamori W., Adachi S. (2020). Rice Cultivar Takanari Has Higher Photosynthetic Performance Under Fluctuating Light Than Koshihikari, Especially Under Limited Nitrogen Supply and Elevated CO_2_. Front. Plant Sci..

[40] Matthews J.S., Vialet-Chabrand S., Lawson T. (2018). Acclimation to Fluctuating Light Impacts the Rapidity of Response and Diurnal Rhythm of Stomatal Conductance. Plant Physiol..

[41] Long S.P., Bernacchi C.J. (2003). Gas exchange measurements, what can they tell us about the underlying limitations to photosynthesis? Procedures and sources of error. J. Exp. Bot..

[42] Loriaux S.D., Avenson T.J., Welles J.M., Mcdermitt D.K., Eckles R.D., Riensche B., Genty B. (2013). Closing in on maximum yield of chlorophyll fluorescence using a single multiphase flash of sub-saturating intensity. Plant Cell Environ..

[43] Harbinson J., Genty B., Baker N.R. (1989). Relationship between the Quantum Efficiencies of Photosystems I and II in Pea Leaves. Plant Physiol..

[44] Krall J.P., Edwards G.E. (1992). Relationship between photosystem II activity and CO_2_ fixation in leaves. Physiol. Plant..

[45] Yin X., Struik P.C., Romero P., Harbinson J., Evers J.B., Van Der Putten P.E.L., Vos J. (2009). Using combined measurements of gas exchange and chlorophyll fluorescence to estimate parameters of a biochemical C photosynthesis model: A critical appraisal and a new integrated approach applied to leaves in a wheat (*Triticum aestivum*) canopy. Plant. Cell Environ..

[46] Harley P.C., Loreto F., Di Marco G., Sharkey T.D. (1992). Theoretical considerations when estimating the mesophyll conductance to CO_2_ flux by analysis of the response of photosynthesis to CO_2_. Plant Physiol..

[47] von Caemmerer S., Farquhar G.D. (1981). Some relationships between the biochemistry of photosynthesis and the gas exchange of leaves. Planta.

[48] Farquhar G.D., von Caemmerer S., Berry J.A. (1980). A biochemical model of photosynthetic CO_2_ assimilation in leaves of C3 species. Planta.

[49] Warren C.R., Dreyer E. (2006). Temperature response of photosynthesis and internal conductance to CO_2_: Results from two independent approaches. J. Exp. Bot..

[50] Eyland D., van Wesemael J., Lawson T., Carpentier S. (2021). The impact of slow stomatal kinetics on photosynthesis and water use efficiency under fluctuating light. Plant Physiol..

[51] Hermida-Carrera C., Kapralov M.V., Galmés J. (2016). Rubisco Catalytic Properties and Temperature Response in Crops. Plant Physiol..

